# Identification of Immunity-Related Genes in *Dialeurodes citri* against Entomopathogenic Fungus *Lecanicillium attenuatum* by RNA-Seq Analysis

**DOI:** 10.1371/journal.pone.0162659

**Published:** 2016-09-19

**Authors:** Shijiang Yu, Lili Ding, Ren Luo, Xiaojiao Li, Juan Yang, Haoqiang Liu, Lin Cong, Chun Ran

**Affiliations:** Citrus Research Institute, Southwest University/Chinese Academy of Agricultural Sciences, National Engineering Research Center for Citrus, Chongqing 400712, China; Fujian Agriculture and Forestry University, CHINA

## Abstract

*Dialeurodes citri* is a major pest in citrus producing areas, and large-scale outbreaks have occurred increasingly often in recent years. *Lecanicillium attenuatum* is an important entomopathogenic fungus that can parasitize and kill *D*. *citri*. We separated the fungus from corpses of *D*. *citri* larvae. However, the sound immune defense system of pests makes infection by an entomopathogenic fungus difficult. Here we used RNA sequencing technology (RNA-Seq) to build a transcriptome database for *D*. *citri* and performed digital gene expression profiling to screen genes that act in the immune defense of *D*. *citri* larvae infected with a pathogenic fungus. *De novo* assembly generated 84,733 unigenes with mean length of 772 nt. All unigenes were searched against GO, Nr, Swiss-Prot, COG, and KEGG databases and a total of 28,190 (33.3%) unigenes were annotated. We identified 129 immunity-related unigenes in transcriptome database that were related to pattern recognition receptors, information transduction factors and response factors. From the digital gene expression profile, we identified 441 unigenes that were differentially expressed in *D*. *citri* infected with *L*. *attenuatum*. Through calculated Log_2_Ratio values, we identified genes for which fold changes in expression were obvious, including cuticle protein, vitellogenin, cathepsin, prophenoloxidase, clip-domain serine protease, lysozyme, and others. Subsequent quantitative real-time polymerase chain reaction analysis verified the results. The identified genes may serve as target genes for microbial control of *D*. *citri*.

## Introduction

*Dialeurodes citri Asahmaed* (*D*. *citri*) belongs to the family Aleyrodidae, of the order Hemiptera. This important, widely spreading pest is found in the world’s citrus producing areas. It originates from Southeast Asia and has been found in Asia, South and North America, and Europe [1, 2]. *D*. *citri* has a piercing/sucking mouthpart, enabling them to suck the branches and leaves of citrus, which will cause leaf and fruit abscission. More seriously, *D*. *citri* can secrete honeydew, which can adsorb dust in the air and provide nutrition for some fungi. This leads to the occurrence of sooty mold and seriously affects photosynthesis and the quality of citrus fruits. In the state of Florida in the USA, *D*. *citri* was once the dominant pest for citrus trees [3]. In Oceania, *D*. *citri* was detected for the first time in New Zealand in 2000 [4], and it spread swiftly to other citrus-producing regions, leading to a reduction in output by about 90% [5]. The citrus-producing regions in Chongqing, China, such as Changshou, Tongnan, Tongliang, and Nanchuan, are at risk for large-scale outbreaks of *D*. *citri*, which bring much trouble to growers. At present, the main method for preventing *D*. *citri* infection is chemical control. However, it is difficult for a chemical pesticide to effectively control an outbreak of *D*. *citri* infection in a field. In addition, excessively frequent chemical spraying may lead to environmental pollution, pesticide residue, and drug resistance [6–8]. Therefore, an environmentally, friendly and effective control strategy for *D*. *citri* is needed.

*Lecanicillium attenuatum* (*L*. *attenuatum*), a member of the order Hypocreales, is an important entomopathogenic fungus. *L*. *attenuatum* parasitizes various pests, such as whiteflies, scale insects, aphids, and nematodes [9–12]. Recent research suggested that the fungus can also parasitize fungi, causing cucumber powdery mildew [13]. At present, *L*. *attenuatum* has been separated from crops in many countries [14–17]. Our laboratory successfully separated the fungus from *D*. *citri* corpses collected in a citrus nursery of Chongqing. The obtained fungus showed a strong infection ability in *D*. *citri*.

However, it is difficult for pathogenic fungi to infect *D*. *citri*. Pests, similar to those higher animals, have a complete immune defense system, which decreases greatly the lethality of pathogenic fungi. It is well known that insects possess three major lines of immune defense: the body wall, cell-mediated immunity, and humoral immunity. The body wall of insects is their first barrier against invaders. The main functional parts of the body wall are chitin, protein, wax, and some recognition factors. The cellular immunity of the insect relies mainly on the ability of the hemocytes in the body to enwrap and devour the invaders and antigens. In addition, hemocytes are also involved in wound healing and blood coagulation, acting to prevent pathogenic microorganisms from entering the body through a wound [18]. Gillespie et al. found that, after treatment by the Metarhizium acridum, the desert locust shows a sharp rise in the number of hemocytes and begins, playing a rold in the immune response [11]. This means the hemocytes respond rapidly, playing an important role in cellular immunity. The insect humoral immune response includes melanization, lysozyme, antimicrobial peptides (AMPs), lectins, antiviral factors (AvFs), and proteinase inhibitors (Pis). In case of microorganism infection, the insect can activate the cellular and humoral immune responses through four steps [19]: 1) identify the invaders. The insect has special pattern recognition receptors (PRRs), such as peptidoglycan recognition proteins (PGRPs), thioester-containing proteins (TEPs), gram-negative binding proteins (GNBPs), scavenger receptors (SCRs), C-type lectins (CTLs), and galectin (GALE), that can identify the external pathogenic microorganisms, leading to a downstream immune response [20]; 2) an extracellular cascade reaction to activate serine proteases and remove serine protease inhibitor; 3) amplification of the signal of infection or removal of the false alarms; 4) stimulation of the transcription of the effecting factor through a signal transduction pathway to produce the immune response. The AMPs are mainly subjected to the Toll pathway, Imd pathway, and Jas/Stat signal transduction pathway.

At present, genes and proteins related to the immune response is mainly studied in a model insect. However, there have been few studies on the identification of genes in *D*. *citri* that are responsible for the immune response to *L*. *attenuatum* infection. Our analyses of the transcriptome of *D*. *citri* (no reference genome) and differential expression of genes in the digital gene expression (DGE) profile provide information on immunity-related genes of *D*. *citri* and a theoretical basis for research into the molecular mechanism of the immunity of *D*. *citri* against pathogenic fungi. With gene function annotation, it is possible to find the major genes responsible for the defense of *D*.*citri* against *L*. *attenuatum*, and thus, the limitations and blindness of research on a single gene are reduced. This research will benefit the biological control of insects using pathogenic fungi.

## Materials and Methods

### Fungus culture and conidia suspension preparation

*L*. *attenuatum* strain TL001 was cultured on potato dextrose agar (PDA) plates at 25°C and 80% humidity. Conidia (spores) used for infection were harvested from 3–4 weeks old cultures by scraping the surface of the mycelia with sterile cell scrapers into sterile deionized water containing 0.1% Tween-80. Conidia were separated from other mycelial structures over a sterile funnel packed with autoclaved glass wool, washed twice with ddH_2_O by centrifugation at 4,000 rpm, counted, and diluted to 1×10^8^ spores/ml. Freshly prepared conidia were used for all experiments.

### Collection of D. citri and RNA extraction

*D*. *citri* eggs, nymphs of all ages, and adults were collected in the net house of *D*. *citri* and transferred to a 1.5-ml EP tube in equal proportions. The EP tube was numbered #1, frozen in liquid nitrogen, and stored at -80°C.

The citrus leaves to which the *D*. *citri* larvae were attached were collected and washed with clear water. These leaves were separated into two groups. One group was treated with the spore suspension for 3 days, whereas the other group, as the control group, was sprayed with sterile water. The successful infection of *D*. *citri* larvae with *L*. *attenuatum* was confirmed under a dissecting microscope by observation that the attached spores on the cuticle of larvae had germinated and enwrapped pests’ bodies. *D*. *citri* larvae in the control and treatment groups were picked and transferred to two 1.5-ml EP tubes, numbered #2 and #3, respectively. The contents of the EP tubes were frozen with liquid nitrogen and stored at -80°C.

Using the RNA Isolater Total RNA Extraction Reagent (Vazyme, China) method, total RNA was extracted from samples #1, #2 and #3. The genomic DNA in the total RNA was removed using DNase I (Invitrogen, USA). The quality of the extracted RNA was evaluated through 1% agarose gel electrophoresis, and the concentration was determined with an Nanodrop 2000N spectrophotometer (Thermo Fisher Scientific, USA), recorded as optical density ratios OD260/OD280 and OD260/OD230.

### Construction and sequencing of cDNA library

The RNA (#1) with a poly(A) tail was purified with magnetic beads containing polynucleotide T and ruptured with buffer solution. Enriched poly(A) RNA of each sample was fragmented into 200–700 nt pieces with RNA Fragmentation Reagents. Then, the first-strand cDNA was synthesized with reverse transcriptase and arbitrary primers. Next, the second-strand cDNA was synthesized with DNA polymerase I and RNA enzyme. The double-stranded DNA was modified with Klenow fragment and T4 polynucleotide kinase successively. A base A was added on the 3'→5' exonuclease of the Klenow fragment. Then the base was connected to the corresponding adaptor using T4 ligase. Finally, fragments with around 200bp length were purified with QiaQuick GelPurify Kit (Qiagen, Hilden, Germany), and used as templates for PCR amplification to create the cDNA library. The library was paired-end sequenced using PE100 strategy on Illumina HiSeqTM 2500 (Illumina, San Diego, CA, USA) in Biomarker Technologies (Beijing, China).

### Assembly and annotation of transcriptomes

Trinity (http://trinityrnaseq.sourceforge.net/) software was applied to perform the *de novo* assembly for the filtered high-quality data. The used parameters were as follow: min_glue = 2, V = 10, edge-thr = 0.05, min_kmer_cov = 2, kmer size = 25, path_reinforcement_distance = 80, and group_pairs_distance = 250. The other parameters were set as the default. The data samples were merged and assembled, and contigs were obtained through overlap of the assembled sequences. Then the contigs were clustered according to the paired-end information of sequences and similarity of contigs. Local assembly was conducted to generate transcripts. The longest transcript in each local region was selected for use as a unigene. The following parameters were used to ensure a high quality of assembly: a minimum of 95% identity, a minimum of 35 overlapping bases, a minimum of 35 scores and a maximum of 25 unmatched overhanging bases at sequence ends. The consensus cluster sequences and singletons make up the final unigene dataset. For functional annotations, we first searched all unigene sequences against various protein databases such as Nr, SwissProt, COG, and KEGG using BLASTX, and then searched nucleotide database Nt using BLASTN, with an E-value cut-off of 10^−5^. For inconsistencies between the unigene alignment results in different databases, priority was given to the nr, Swiss-Prot, KEGG, and COG data sequentially. Unigenes with no matched data in any of these databases were analyzed with ESTScan software to predict the coding region and direction of the sequence.

### Analysis on the digital gene expression profile

According to data in the constructed cDNA library, differences in mRNA expression between samples #2 and #3 were identified using the digital gene expression (DGE) profile. Gene expression was quantified as reads per kb per million reads (RPKM) [21]. According on Audic et al.'s detection method for differentially expressed genes based on sequencing [22], a strict algorithm was adopted to screen the differentially expressed genes. Those with a false discovery rate (FDR) ≤0.001 and |fold change| ≥2 were considered to be differentially expressed. Combined with the function annotation of differentially expressed genes, the pathway (KEGG) and gene ontology (GO) enrichment analysis as well as the pattern clustering of differentially expressed genes were performed. Fisher's exact test was applied in the enrichment analysis. The results were adjusted using the Bonferroni correction method. Thus, the pathways with obviously enriched differentially expressed genes and GO functional categories were obtained for further analysis.

### qRT-PCR veritification on DGE

*D*. *citri* larvae of the same age were collected and sprayed with the spore suspension of *L*. *attenuatum* at the concentration of 1×10^8^ spores/mL. The infection period lasted 5 days. A proper amount of *D*. *citri* was picked out every day, frozen with liquid nitrogen, and preserved at -80°C. *D*. *citri* larvae treated with sterile water were used as the control. The primers for differentially expressed genes and reference genes were designed using Primer Premier 5 software (S1 Table). The total RNA from the six treatments above was extracted separately, and 1 μg was used in the reverse transcription with HiScript II Q RT SuperMix for qPCR with gDNA wiper (Vazyme, China), followed by real-time fluorescent quantitative PCR with ChamQ SYBR qPCR Master Mix (Vazyme). The reaction was carried out on an ABI 7500 Fast Real-Time PCR System (Applied Biosystems, USA). Relative gene expression was calculated using the Pfaffl method [23]. Both *D*. *citri α-tubulin* and *β-actin* were used as reference genes. The analytic software for qPCR was 7500 softwear v2.0.6. SPSS Statistics v19.0.0 software was used to perform independent sample t-tests (*P*<0.05).

## Results

### Transcriptome sequencing and assembly

Through transcriptome sequencing (runs accession number: SRR2980521), we found a total of 24,071,734 reads. The mean CycleQ20 value of the samples reached 100.00%, and the base Q30 was 81.41%, suggesting that the sequencing was reliable. All high-quality reads were assembled *de novo* into 2,318,371 contigs (Table 1) with a mean length of 82 nt. Contigs were clustered according to the paired-end information of sequences and similarity of contigs and then assembled into 119,428 transcripts with the mean length of 952 nt. Among them, 31,748 transcripts (26.59%) had a length greater than 1,000 nt. These transcripts were finally assembled into 84,733 unigenes, with a mean length of 772 nt. Among them, 16,602 unigenes (19.6%) had a length greater than 1,000 nt. The N50 lengths of the contigs, transcripts, and unigenes were 98 nt, 1,809 nt and 1,320 nt, respectively. Regarding the length distribution of transcripts and unigenes of *D*. *citri*, most were 200–300 nt, followed by 300–500 nt, with the smallest group being sequences longer than 2,000 nt.

**Table 1:** cDNA library assembly for *D*. *citri*.

Length range	Contigs	Transcripts	Unigenes
200–300	2,251,642(97.12%)	32,280(26.97%)	26,903(31.75%)
300–500	28,533(1.23%)	30,365(25.43%)	24,241(28.61%)
500–1000	19,900(0.86%)	25,107(21.02%)	16,987(20.05%)
1000–2000	10,759(0.46%)	16,823(14.09%)	9,521(11.24%)
2000+	7,537(0.33%)	14,925(12.50%)	7,081(8.36%)
Total number	2,318,371	119,428	84,733
Total length	191,192,105	113,647,937	65,399,254
N50 length	98	1,809	1,320
Mean length	82.47	951.6	771.83

### Function annotation of unigenes

Among the 84,733 assembled unigenes, 28,190 unigenes had been annotated, as found through the sequence alignment on the NCBI (National Center for Biotechnology Information database) website. Specifically, 11,516 unigenes were annotated in the COG database, 17,081 in the GO database, 19,537 in the Swiss-Prot database, 9,387 in the KEGG database, and 27,746 in the Nr database (Table 2).

**Table 2:** Functional annotation of the *D*. *citri* transcriptome.

Anno_Database	Annotated_Number	300< = length<1000	length> = 1000
COG_Annotation	11516	4977	4405
GO_Annotation	17081	7071	6800
KEGG_Annotation	9387	3855	3727
Swissprot_Annotation	19537	8050	8280
nr_Annotation	27746	12159	10355
All_Annotated	28190	12396	10380

**Fig 1:**
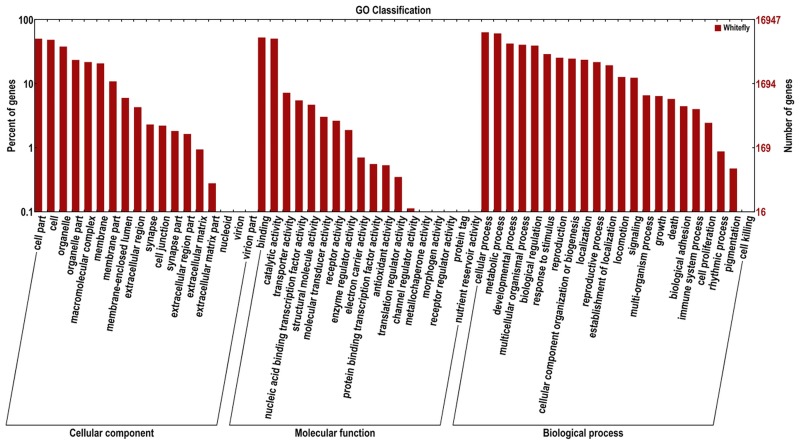
Functional annotation of assembled sequences based on gene ontology (GO) categorization. GO analysis was performed at level two for three main categories (cellular component, molecular function, and biological process).

It was observed in GO analysis that 17,081 unigenes were named as 148,314 GO terms, of which most genes included more than one GO term. GO analysis was used mainly to predict the functions of *D*. *citri* proteins. Generally, GO terms are classified into three categories (Fig 1): biological process, molecular function, and cellular component. In our analysis, most of the GO terms participated in biological process (90,520, accounting for 61.03% of the total), followed by molecular function (32,827, 22.13%), and cellular component (24,967, 16.83%). The three largest sub-categories of GO terms were cellular process (10,798 GO terms) and metabolic process (10,357 GO terms) in biological process and binding (8,959 GO terms) in the molecular function.

**Fig 2:**
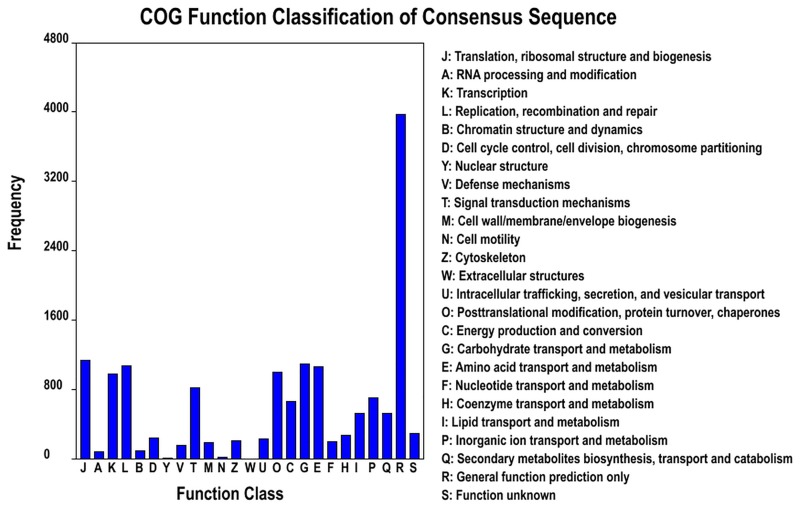
Histogram representation of Clusters of Orthologous Groups (COG) classification.

We also used COG classifications to analyze the putative protein functions. In total, 11,516 unigenes were functionally classified into 25 COG categories (Fig 2). The largest category was “General function prediction only” (3,976, 35%), followed by “Translation, ribosomal structure and biogenesis” (1,134, 9.9%), “Carbohydrate transport and metabolism” (1,097, 9.5%), “Replication, recombination and repair” (1,067, 9.3%), “Amino acid transport, metabolism” (1,057, 9.2%). There were only a few of unigenes taking part in “Nuclear structure” (7 unigenes), “Cell motility” (20 unigenes), “RNA processing and modification” (81 unigenes), and “Chromatin structure and dynamics” (94 unigenes). No unigenes took part in Extracellular structures.

**Fig 3:**
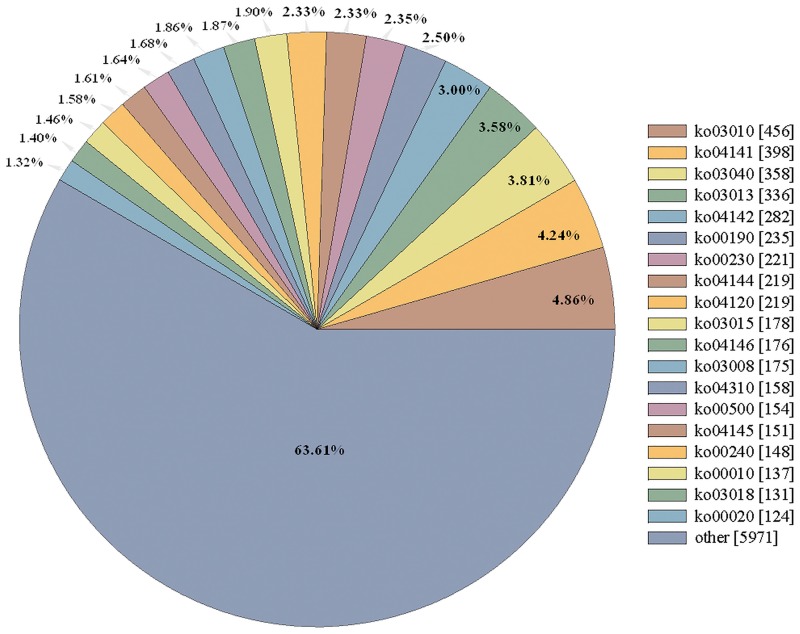
Pie chart representation of the distribution of functional annotations of Kyoto Encyclopedia of Genes and Genomes (KEGG). ko03010: Ribosome; ko04141: Protein processing in endoplasmic reticulum; ko03040: Spliceosome; ko03013: RNA transport; ko04142: Lysosome; ko00190: Oxidative phosphorylation; ko00230: Purine metabolism; ko04120: Ubiquitin mediated proteolysis; ko04144: Endocytosis; ko03015: mRNA surveillance pathway; ko04146: Peroxisome; ko03008: Ribosome biogenesis in eukaryotes; ko04310: Wnt signaling pathway; ko00500: Starch and sucrose metabolism; ko04145: Phagosome; ko00240: Pyrimidine metabolism; ko00010: Glycolysis / Gluconeogenesis; ko03018: RNA degradation; ko00020: Citrate cycle (TCA cycle).

There were 9,387 unigenes that had been annotated and classified in KEGG database, distributed over 208 pathways (Fig 3). The numbers of unigenes involved in different pathways varied. Those pathways with less than 100 unigenes were classified into one category (others), and the remaining 3,416 pieces of unigenes were classified into 19 categories. The ribosome pathway (ko03010) was the largest category with 456 pieces of unigenes. The pathways of protein processing in the endoplasmic reticulum (ko04141, 398 pieces), spliceosome (ko03040, 358 pieces), and RNA transportation (ko03013, 336 pieces) also involved many unigenes.

### Identification of immunity-related genes

**Fig 4:**
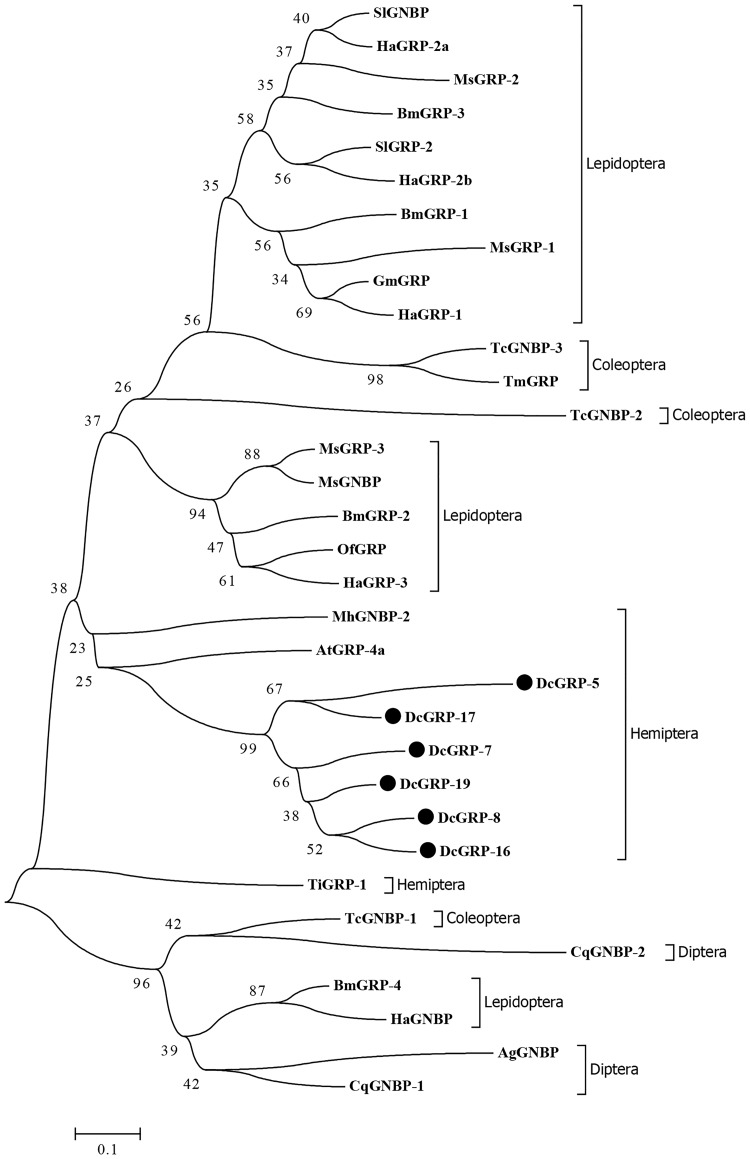
Phylogenetic analysis of β-1,3-glucan recognition proteins (βGRPs) from *D*. *citri* and other insect species. The used amino acid sequences are from *Dialeurodes citri* (Dc), *Triatoma infestans* (Ti), *Anasa tristis* (At), *Bombyx mori* (Bm), *Maconellicoccus hirsutus* (Mh), *Anopheles gambiae* (Ag), *Tribolium castaneum* (Tc), *Tenebrio molitor* (Tm), *Ostrinia furnacalis* (Of), *Spodoptera litura* (Sl), *Manduca sexta* (Ms), *Galleria mellonella* (Gm), *Helicoverpa armigera* (Ha), *Culex quinquefasciatus* (Cq).

By searching the transcriptome, we preliminarily identified 129 pieces of immunity-related unigenes, involving pattern recognition receptors, signal transduction factors, response factors, and so on (Table 3). These genes play important roles in endogenous and exogenous immunoreactions of *D*. *citri*. For example, prophenoloxidase (PPO) can participate in melanization; lysozyme can be capable of dissolving cell walls of fungi and Gram-positive bacteria; and the antimicrobial peptide and clip domain serine protease also perform functions related to humoral immunity when a pathogen invasion occurs. In terms of the pattern recognition receptors, we totally identified a total of 49 unigenes including 1 PGRP, 6 GNBPs, 19 βGRPs, 2 TEPs, 8 SCRs, 3 CTLs, 5 GALEs, 3 hemocytins, and 2 integrins. These genes play an important role for the insect to recognize external pathogens, triggering the downstream reaction. PGRPs mainly identify the special ingredient in the bacterial cell walls—peptidoglycan—and then trigger the transcription of antibacterial peptide or PPO activation cascade. The protein was originally found in the silkworm, and until now, 12 PGRP genes have been identified [24]. However, in the transcriptome of *D*. *citri*, we identified only one PGRP gene (PGRP—S2). In dealing with the infection of gram negative bacteria and fungi, GNBP/βGRP can identify and combine with the beta-1,3-glucan and trigger PPO cascade. We performed phylogenetic analyses on the complete six genes, *DcβGRP-5*,*7*,*8*,*16*,*17*,*19*, in the sequence of *D*. *citri*, and found they have a close genetic relationship with *AtβGRP-4a* and *MhGNBP-2* in Hemiptera (Fig 4).

**Table 3:** Summary of the immunity-related unigenes identified in *D*. *citri* transcriptome.

Gene Name	Unigene ID	Nucleotide length (bp)	Protein length (aa)	Nr_annotation
**Pattern recognition receptors**				
**Peptidoglycan recognition proteins (PGRPs)**				
DcPGRP	Unigene 51807_c0	467	143	PGRP S2-like protein precursor
**Gram-negative binding proteins (GNBPs)**				
DcGNBP-1	Unigene 61815_c0	322	69	GNBP1
DcGNBP-2	Unigene 44656_c0	274	19	GNBP2
DcGNBP-3	Unigene 309462_c0	329	27	GNBP 2-like protein
DcGNBP-4	Unigene78758_c1	222	57	GNBP 2-like protein
DcGNBP-5	Unigene63821_c0	430	123	GNBP 2-like protein
DcGNBP-6	Unigene70098_c0	598	148	GNBP 2-like protein
**β-1,3-glucan recognition protein (βGRP)**				
DcβGRP-1	Unigene 433117_c0	374	111	βGRP 4a
DcβGRP-2	Unigene 27715_c0	217	42	βGRP 4a
DcβGRP-3	Unigene51664_c0	660	102	βGRP 4a
DcβGRP-4	Unigene61815_c1	389	91	βGRP 4a
DcβGRP-5	Unigene73030_c1	1103	290	βGRP 4a
DcβGRP-6	Unigene59593_c0	485	113	βGRP 4a
DcβGRP-7	Unigene82059_c0	2135	530	βGRP 4a
DcβGRP-8	Unigene78387_c0	1555	416	βGRP 4a
DcβGRP-9	Unigene35662_c0	323	59	βGRP 4a
DcβGRP-10	Unigene65987_c0	558	166	βGRP 4a
DcβGRP-11	Unigene344405_c0	424	125	βGRP 4a
DcβGRP-12	Unigene269973_c0	668	176	βGRP 4a
DcβGRP-13	Unigene63341_c0	574	125	βGRP 4a
DcβGRP-14	Unigene46518_c0	442	146	βGRP 4a
DcβGRP-15	Unigene68139_c0	715	208	βGRP 4a
DcβGRP-16	Unigene80609_c0	1546	280	βGRP 4a
DcβGRP-17	Unigene78758_c0	1522	448	βGRP 4a
DcβGRP-18	Unigene78145_c0	1407	151	βGRP 4a
DcβGRP-19	Unigene80295_c0	1455	410	βGRP 4a
**Thioester-containing proteins (TEPs)**				
DcTEP-1	Unigene5216_c0	245	48	Thioester-containing protein
DcTEP-2	Unigene20936_c0	207	58	Thioester-containing protein
**Scavenger receptors (SCRs)**				
DcSCR-1	Unigene525433_c0	445	77	Scavenger receptor class B member 1 isoform 1
DcSCR-2	Unigene181460_c0	909	245	Scavenger receptor class B member 1
DcSCR-3	Unigene74496_c0	5800	403	Scavenger receptor class B member 1
DcSCR-4	Unigene77847_c0	6047	562	Scavenger receptor class B member 1 isoform 2
DcSCR-5	Unigene79498_c0	4193	521	Scavenger receptor class B member 1 isoform 1
DcSCR-6	Unigene80932_c0	4763	624	Scavenger receptor class B member 1
DcSCR-7	Unigene81170_c0	4728	539	Scavenger receptor class B member
DcSCR-8	Unigene81350_c0	2424	615	Scavenger receptor class B member
**C-type lectins (CTLs)**				
DcCTL-1	Unigene83071_c0	1131	217	C-type lectin-like precursor
DcCTL-2	Unigene281842_c0	410	116	C-type lectin domain-containing protein 141
DcCTL-3	Unigene713818_c0	339	60	C-type lectin domain-containing protein 141
**Galectin (GALE)**				
DcGALE-1	Unigene63173_c0	577	174	Galectin 1
DcGALE-2	Unigene73575_c0	1393	380	Galectin
DcGALE-3	Unigene77505_c0	1182	193	Galectin
DcGALE-4	Unigene78155_c0	1193	366	Galectin
DcGALE-5	Unigene81510_c0	7678	1752	Galectin
**Hemocytin**				
DcHemocytin-1	Unigene42495_c0	576	173	Hemocytin
DcHemocytin-2	Unigene65062_c0	1565	372	Hemocytin
DcHemocytin-3	Unigene67140_c0	987	300	Hemocytin
**Integrin**				
DcIntegrin-1	Unigene77807_c0	3619	1119	Integrin alpha-PS1
DcIntegrin-2	Unigene80101_c0	5509	1657	Integrin alpha-PS2
**Signal transduction**				
**Toll**				
DcToll-1	Unigene72008_c2	598	170	Toll-1
DcToll-2	Unigene138073_c0	919	59	Toll-1
DcToll-3	Unigene82362_c0	4965	460	Protein toll precursor
DcToll-4	Unigene949000_c0	674	72	Similar to toll
DcToll-5	Unigene713993_c0	346	26	Toll
DcToll-6	Unigene700734_c0	612	122	Toll-6
DcToll-7	Unigene78948_c0	5430	1402	Toll-7
DcToll-8	Unigene3070_c0	204	49	Toll-8
DcToll-9	Unigene79212_c0	4729	1013	Toll-like receptor 13-like
DcToll-10	Unigene9735_c0	569	180	Toll-10
DcToll-11	Unigene72008_c0	1528	365	Protein toll precursor
DcToll-12	Unigene163471_c0	1175	353	Protein toll precursor
DcToll-13	Unigene72008_c4	222	32	Protein toll-like
DcToll-14	Unigene78170_c0	2119	513	Protein toll
DcToll-15	Unigene916650_c0	203	56	Toll
DcToll-16	Unigene706924_c0	248	0	Toll-like receptor 3-like
**Evolutionarily conserved signaling intermediate in Toll (ECSIT)**				
DcECSIT	Unigene76455_c0	3595	438	ECSIT isoform 1
**Pelle**				
DcPelle-1	Unigene713608_c0	214	51	Serine/threonine-protein kinase pelle
DcPelle-2	Unigene81953_c1	2771	647	Serine/threonine-protein kinase pelle
DcPelle-3	Unigene940287_c0	245	22	Serine/threonine-protein kinase pelle
DcPelle-4	Unigene955734_c0	214	45	Serine/threonine-protein kinase pelle
DcPelle-5	Unigene254925_c0	1237	301	Serine/threonine-protein kinase pelle
DcPelle-6	Unigene1093739_c0	255	77	Serine/threonine-protein kinase pelle
**Pellino**				
DcPellino	Unigene81554_c1	2229	418	Pellino
**NF-κB**				
DcNF-κB-1	Unigene76830_c0	1952	265	NF-kappa-B-activating protein
DcNF-κB-2	Unigene78848_c0	5921	375	NF-kappa-B inhibitor alpha
DcNF-κB-3	Unigene32609_c0	1874	416	NF-kappa-B-repressing factor
**Clip-domain serine protease (SP)**				
DcSP-1	Unigene65980_c0	708	150	Serine protease
DcSP-2	Unigene76614_c0	1249	392	Serine protease snake-like
DcSP-3	Unigene80903_c0	1403	378	Serine protease snake-like
DcSP-4	Unigene56933_c1	423	68	Serine protease snake-like
DcSP-5	Unigene603158_c0	283	65	Serine protease 22 precursor
DcSP-6	Unigene69896_c0	1839	442	Serine protease gd-like isoform 1
DcSP-7	Unigene2194_c0	355	99	Serine protease P32
DcSP-8	Unigene73010_c0	1270	370	Serine protease snake-like
DcSP-9	Unigene77526_c0	1562	357	Serine protease
DcSP-10	Unigene74736_c0	1369	415	Serine protease snake-like
DcSP-11	Unigene75142_c0	2040	539	Serine protease
DcSP-12	Unigene70828_c1	688	226	Serine protease
DcSP-13	Unigene56933_c0	570	99	Serine protease snake-like
DcSP-14	Unigene188106_c0	320	87	Serine protease
DcSP-15	Unigene439362_c0	558	185	Serine protease
DcSP-16	Unigene62335_c0	413	46	Serine protease
DcSP-17	Unigene80402_c0	1418	402	Serine protease snake-like
DcSP-18	Unigene80015	728	219	Serine protease
DcSP-19	Unigene73992_c0	555	153	Serine protease P58
**Clip-domain serine protease homolog (SPH)**				
DcSPH-1	Unigene74523_c0	3506	411	Prophenoloxidase activating factor
DcSPH-2	Unigene70002_c0	515	131	Prophenoloxidase activating factor
**Serpin**				
DcSerpin-1	Unigene81431_c0	263	19	Serpin 1
DcSerpin-4	Unigene65498_c0	262	39	Serpin 4
DcSerpin-5	Unigene21574_c0	386	52	Serpin 5
DcSerpin-6	Unigene82073_c0	6400	428	Serpin 6
DcSerpin-8	Unigene79484_c0	1177	128	Serpin 8
DcSerpin-9	Unigene65659_c3	834	212	Serpin 9
**Effectors**				
**Prophenoloxidase (PPO)**				
DcPPO-1	Unigene44504_c0	1207	305	Prophenoloxidase, partial
DcPPO-2	Unigene4538_c0	248	71	Prophenoloxidase
DcPPO-3	Unigene538626_c0	239	38	Prophenoloxidase 2
DcPPO-4	Unigene59898_c0	661	95	Prophenoloxidase 2
DcPPO-5	Unigene60106_c0	327	62	Prophenoloxidase VII
DcPPO-6	Unigene69131_c0	2510	709	Prophenoloxidase
DcPPO-7	Unigene78621_c0	2659	702	Prophenoloxidase
DcPPO-8	Unigene827915_c0	409	40	Prophenoloxidase
DcPPO-9	Unigene975841_c0	225	20	Prophenoloxidase 5
DcPPO-10	Unigene29019_c0	617	136	Prophenoloxidase
DcPPO-11	Unigene29019_c1	340	41	Prophenoloxidase 2
DcPPO-12	Unigene304863_c0	347	61	Prophenoloxidase 2
DcPPO-13	Unigene1072959_c0	250	71	Prophenoloxidase subunit 1
DcPPO-14	Unigene312841_c0	240	34	Prophenoloxidase
DcPPO-15	Unigene31435_c0	651	177	Prophenoloxidase-I
DcPPO-16	Unigene33650_c0	288	90	Prophenoloxidase
DcPPO-17	Unigene81686_c0	2641	739	Prophenoloxidase
DcPPO-18	Unigene75007_c0	1830	423	Prophenoloxidase subunit 2
**Lysozyme**				
DcLys-1	Unigene45440_c0	655	154	C-type lysozyme
DcLys-2	Unigene513451_c0	301	79	Lysozyme P-like
DcLys-3	Unigene77954_c0	1412	159	Lysozyme 1-like
DcLys-4	Unigene62670_c0	356	55	Lysozyme P
DcLys-5	Unigene65090_c0	755	143	Lysozyme 3
DcLys-6	Unigene694489_c0	250	61	Lysozyme D
**Antimicrobial peptide (AMP)**				
DcAMP-1	Unigene71869_c0	449	58	Antimicrobial peptide Alo-1
DcAMP-2	Unigene29069_c0	465	107	Antimicrobial peptide Alo-3
DcAMP-3	Unigene69306_c0	260	62	Antimicrobial peptide Alo-3

**Fig 5:**
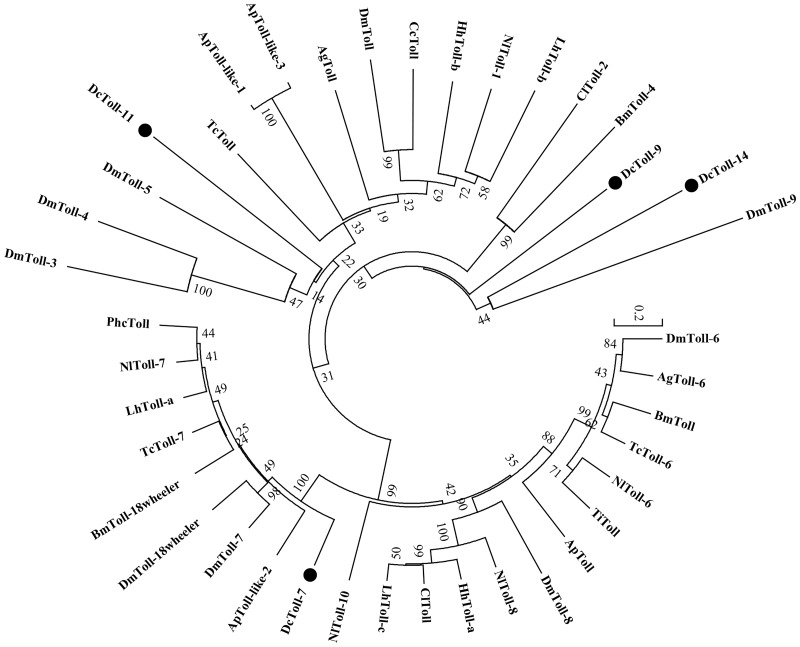
Phylogenetic analysis of Tolls from *D*. *citri* and other insect species. The used amino acid sequences are from *Dialeurodes citri* (Dc), *Nilaparvata lugens* (Nl), *Acyrthosiphon pisum* (Ap), *Graminella nigrifrons* (Gn), *Halyomorpha halys* (Hh), *Cimex lectularius* (Cl), *Lygus hesperus* (Lh), *Triatoma infestans* (Ti), *Drosophila melanogaster* (Dm), *Bombyx mori* (Bm), *Tribolium castaneum* (Tc), *Anopheles gambiae* (Ag), *Pediculus humanus corporis* (Phc), *Ceratitis capitata* (Cc).

Four signal transduction pathways, Toll, Imd, JNK, and JAK/STAT are known to be involved ininsect immunity [25]. However, in *D*. *citri*, we only identified unigenes related to the Toll pathway, including 16 Tolls, 1 ECSIT, 6 Pelle, 1 pellino, and 3 NF-κB genes, and did not find genetic information in the other three pathways. We perform phylogenetic analysis on the Toll protein and found *DcToll-7* has high similarity with other species (Fig 5). In the cascade mediated by serine protease, *D*. *citri* has 19 clip-domain serine proteases (clip-domain SP), 2 serine protease homologs (SPHs), and 6 serpins. Among them, *DcSPH-1* and *DcSPH-2* are prophenoloxidase-activating factors, which can catalyze conversion of PPO into PO (phenoloxidase). Serpin can negatively control the PPO activation, competing for PPO with SP.

**Fig 6:**
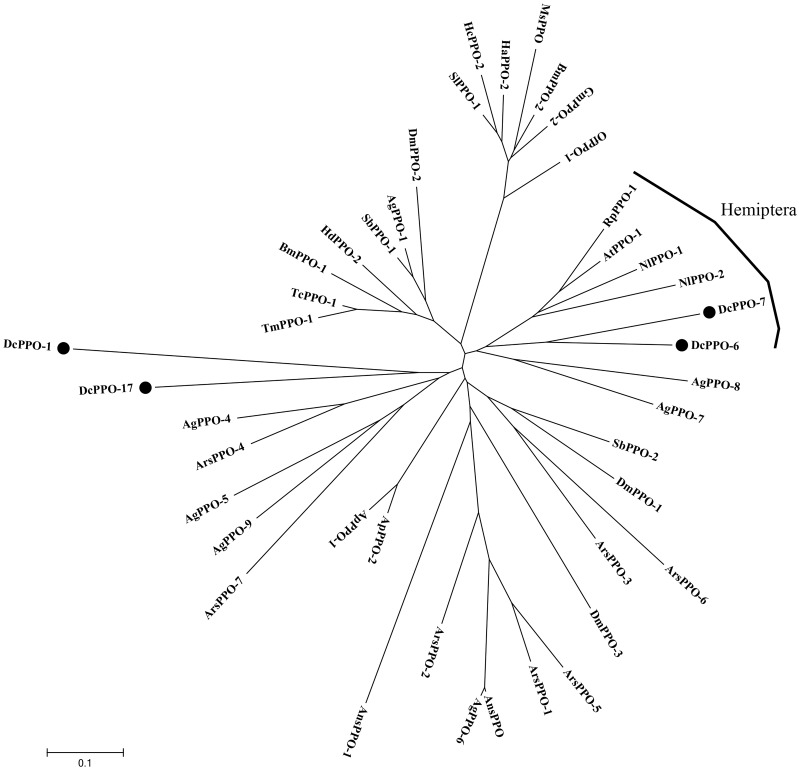
Phylogenetic analysis of prophenoloxidases (PPOs) from *D*. *citri* and other insect species. The used amino acid sequences are from *Dialeurodes citri* (Dc), *Tenebrio molitor* (Tm), *Anasa tristis* (At), *Riptortus pedestris* (Rp), *Acyrthosiphon pisum* (Ap), *Drosophila melanogaster* (Dm), *Bombyx mori* (Bm), *Anopheles gambiae* (Ag), *Tribolium castaneum* (Tc), *Spodoptera litura* (Sl), *Sarcophaga bullata* (Sb), *Ostrinia furnacalis* (Of), *Manduca sexta* (Ms), *Holotrichia diomphalia* (Hd), *Hyphantria cunea* (Hc), *Helicoverpa armigera* (Ha), *Galleria mellonella* (Gm), *Anopheles stephensi* (Ans), *Armigeres subalbatus* (Ars), *Nilaparvata lugens* (Nl).

As immune factors of *D*. *citri*, we identified 18 PPO unigenes, 6 lysozyme unigenes, and 3 AMPs. Most of the insects contained two kinds of PPOs, in the form of dimmers. Through clustering analysis, we found *DcPPO-6* and *DcPPO-7* with PPO of Hemiptera, and *DcPPO1* and *DcPPO-17* had a more distant relationship with other species (Fig 6). This may suggest that *D*. *citri* has more than two kinds of PPO genes. AMP includes cecropin, defensin, attacin, gloverin, and so on. *D*. *citri* has 3 AMP genes with sequences that are too short to permit identification of the type of AMP.

### Expression profile analysis

**Fig 7:**
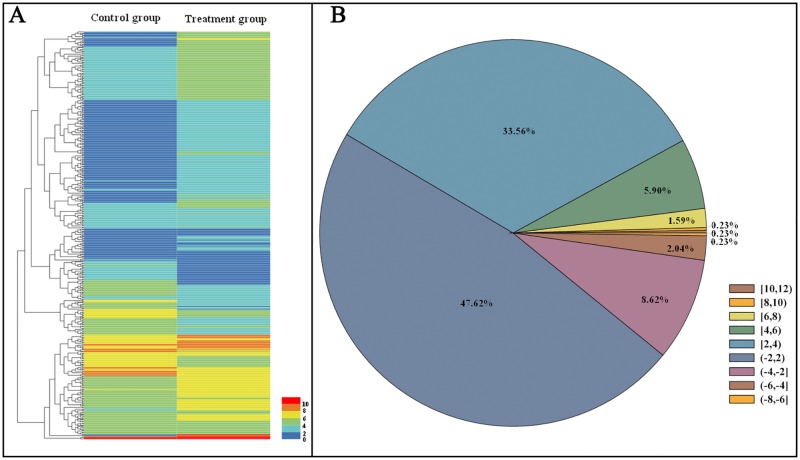
Clustering analysis (A) and quantitative analysis (B) of differentially expressed genes in *D*. *citri* after treatment with *L*. *attenuatum*. The numbers beside the bar mean the values of Log_2_FC.

We obtained the RPKM values for unigenes, and through further calculation of Log_2_ fold changes [Log_2_Ratio(treatment group RPKM/control group RPKM)], we found 441 differentially expressed unigenes in control group and treatment group, among which 313 unigenes had been annotated (Data Accessibility) in the transcriptome database. The clustering analysis of differentially expressed genes suggested that Log_2_FC values of most genes in the treatment group were positive (Fig 7A). Namely, these genes were upregulated in comparison to the control group. It was found in statistical analysis of differentially expressed genes that the genes with Log_2_FC values in the range of -2 to 2 were in the majority (210 pieces, 47.62%; Fig 7B). Genes with Log_2_Ratio values in the range of 2 to 4, ranked second with 148 pieces (33.56%). Generally, only small numbers of differentially expressed genes were downregulated. There were 38 genes (8.62%) with Log_2_FC values of -4 to -2, and 9 genes (2.04%) with Log_2_FC values of -6 to -4. In our two experimental groups, only one gene was upregulated by more than 10-fold, and its gene ID was *unigene78104_c1*. The gene ID of the most downregulated gene (by -6.38-fold) was *unigene80382_c2*.

We analyzed 24 genes that may participate in the response against infection by *L*. *attenuatum* (Table 4). Among them, function annotation identified that the cuticle protein was the highest upregulated gene with a Log_2_Ratio value of 10.04, followed by the vitellogenin gene. This implies that the two proteins may play roles in the response of *D*. *citri* against infection by *L*. *attenuatum* directly or indirectly. Regarding the melanization, we found three PPO genes and three clip-domain SP genes. Among them, the expression difference of *DcSP-3* was 2.51-fold, and those of the two PPO genes were 1–2-fold. In addition, we found two lysozyme unigenes with fold changes of 2.47 and 2.60. Regarding the metabolic reaction process of insects, we examined the expression of cathepsin B.

**Table 4:** Differentially expressed unigenes likely involved in the antifungal response of *D*. *citri* after treatment with *L*. *attenuatum*.

Gene Name	Unigene ID	CG-RPKM	TG-RPKM	Log_2_Ratio (TG/CG)	Up-Down	FDR	Nr_annotation
DcCP-1	Unigene78104_c1	1	1652	10.04	Up	0	Cuticle protein
DcVg-1	Unigene73224_c1	0	77	6.65	Up	0	Vitellogenin
DcVg-2	Unigene77391_c0	9	640	6.33	Up	0	Vitellogenin
DcVg-3	Unigene80752_c1	0	52	6.09	Up	1.43E-13	Vitellogenin
DcVg-4	Unigene59142_c0	0	43	5.82	Up	2.73E-11	Vitellogenin
DcCP-2	Unigene68271_c0	4	213	5.75	Up	0	Cuticle protein
DcVg-5	Unigene73224_c0	1	75	5.59	Up	0	Vitellogenin
DcVg-6	Unigene73224_c2	2	106	5.49	Up	0	Vitellogenin 1
DcVg-7	Unigene78645_c0	0	33	5.45	Up	1.30E-08	Vitellogenin
DcVg-8	Unigene82046_c0	109	2693	4.94	Up	0	Vitellogenin
DcHp-1	Unigene82372_c0	0	21	4.82	Up	3.56E-05	Hypothetical protein
DcVg-9	Unigene78104_c0	4	85	4.43	Up	4.44E-16	Vitellogenin-1
DcH70-1	Unigene693684_c0	1	22	3.86	Up	0.00024	Heat shock cognate 70
DcLys-4	Unigene62670_c0	99	484	2.6	Up	0	Lysozyme
DcSP-3	Unigene80903_c0	8	40	2.51	Up	0.00023	Clip-domain serine protease
DcLys-2	Unigene513451_c0	63	282	2.47	Up	1.11E-15	Lysozyme
DcCatB-1	Unigene78697_c0	152	522	2.09	Up	3.26E-14	Cathepsin B
DcCatB-2	Unigene76462_c0	240	781	2.02	Up	2.43E-14	Cathepsin B
DcPPO-7	Unigene78621_c0	103	306	1.88	Up	7.25E-10	Prophenoloxidase
DcLRTP-1	Unigene71096_c0	49	109	1.46	Up	0.001712	Leucine-rich transmembrane protein
DcSP-18	Unigene80015_c0	117	239	1.35	Up	0.000245	Clip-domain serine protease
DcPPO-6	Unigene69131_c0	98	183	1.21	Up	0.00537	Prophenoloxidase
DcPPO-18	Unigene75007_c0	542	192	-1.17	Down	0.00233	Prophenoloxidase 2
DcSP-9	Unigene77526_c0	700	194	-1.53	Down	7.80E-07	Clip-domain serine protease

Note: CG-RPKM: reads per kb per million reads of cDNA library generated from control group of *D*. *citri*.; TG-RPKM: reads per kb per million reads of cDNA library generated from treatment group of *D*. *citri*.; Log_2_Ratio (TG/CG): Log_2_ Fold Change = Log_2_Ratio(treatment group RPKM/control group RPKM); FDR: false discovery rate.

Pathway analysis of upregulated genes of interest suggested that the differentially expressed genes were mainly involved in two pathways, the lysosome (ko04142) and MAPK signaling pathways (ko04010). In the lysosome pathway, 9 pieces of unigenes exhibited differential expression, with 8 upregulated and 1 downregulated. All of these genes are crucial to lysosome phagocytosis and immunoreactions. The MAPK signaling pathway plays the role of signal transduction in the processes of stress adaptation and inflammatory response. For this pathway, there were two pieces of differentially expressed genes, both of which were related to heat shock cognate 70.

### qRT-PCR analysis of differentially expressed genes

To verify the accuracy of fold changes in the digital gene expression profile and investigate the dynamic tendency of key gene expression with infection by *L*. *attenuatum*, we used qRT-PCR to analyze key genes in the response of *D*. *citri* to infection for 1–5 days.

**Fig 8:**
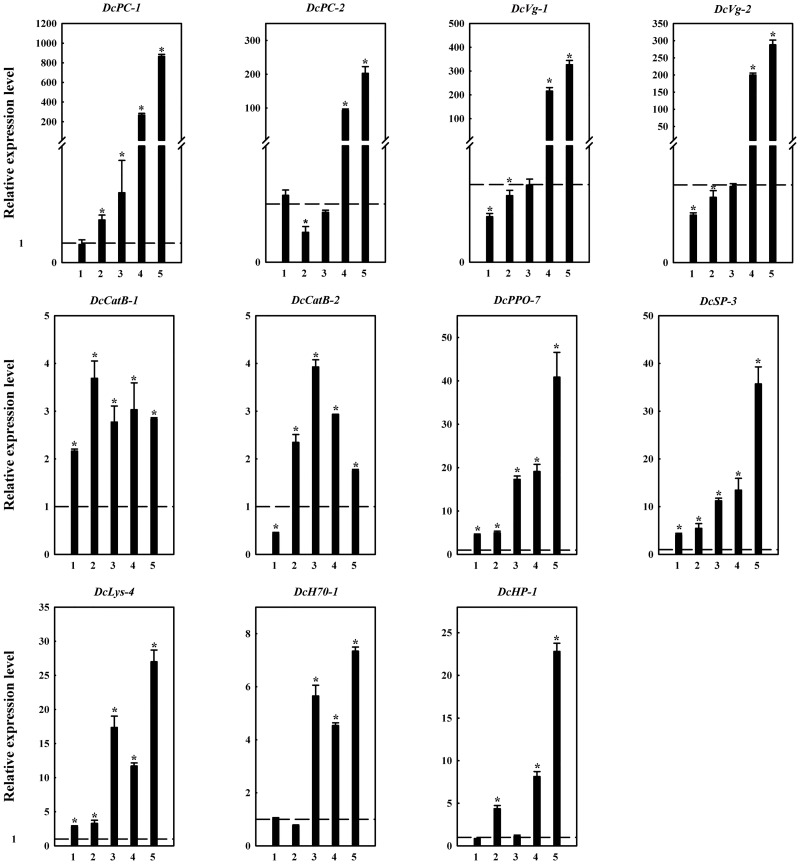
qRT-PCR–based verification and analysis of the differential expression of the identified genes. Data are shown as means of three replicates ± standard deviation (SD). Asterisks above bars indicate significance differences between the treatment and control groups (*t-*test, *P*<0.05). The expression levels of unigenes in the control group (CG) are marked with a dashed line at Y = 1.0. The abscissa values 1, 2, 3, 4, 5 mean the treatment times (day) of *D*.*citri* infected by *L*. *attenuatum*.

qRT-PCR analysis was conducted for the two pieces of cuticle protein unigenes most upregulated. After 1 day of infection, no significant change (*P*<0.05) in their expression was observed compared with that in the control group (CG) treated with clear water. However, by the second day of infection, *DcCP-1* exhibited increased expression (Fig 8). The relative expression change after 2 days was 2.20-fold, and that after 3 days was 3.60-fold. Unexpectedly, the gene expression increased sharply on the fourth day, with 267.83-fold upregulation and 865.54-fold upregulation on the fifth day. The gene *DcCP-2* exhibited obvious downregulation (*P*<0.05) on the second day and rebounded on the third day. On the fourth day, expression of the gene increased sharply as for *DcCP-1*, to 92.87-fold, and further increased to 202.63-fold on the fifth day.

We verified the two upregulated vitellogenin genes, *DcVg-1* and *DcVg-2*, and found that they showed the same expression tendency (Fig 8). During the first 2 days of infection, their expression was inhibited compared to that in the control group (*P*<0.05). On the third day, their expression levels were comparable to those in the control group, and after infection for 4 days, obvious upregulation of the two genes occurred (215.90-fold and 199.98-fold respectively). On the fifth day, these genes were upregulated by 327.19-fold and 287.84-fold, respectively. Thus, expression of the two vitellogenin genes was positively correlated with the infection time.

Among the genes participating in the lysosome pathway, two cathepsin B genes, *DcCatB-1* and *DcCatB-2*, were analyzed through qPCR. *DcCatB-1* was upregulated significantly (*P*<0.05) by 2.17-fold by the first day of infection and continued to be upregulated on days 2–5, with a peak fold change value of 3.69-fold on the second day (Fig 8). Expression of *DcCatB-2* was inhibited on the first day, with a 0.43-fold difference from that in the control group. However, it was upregulated obviously on the second day and reached maximum upregulation by 3.93-fold on the third day. On the fourth and fifth days, its expression decreased, which might be caused by depletion of the enzyme in the latter stage.

For the melanization pathway, we tested the expression levels of *DcPPO-7* and *DcSP-3* (Fig 8). The results indicated that these two genes were significantly upregulated (*P*<0.05) on the first day of infection by 4.40-fold and 4.12-fold, respectively, and by 40.88-fold and 35.71-fold, respectively, by the fifth day. During melanization, serine protease can activate PPO and convert it to PO, which participates in the generation of melanin. This is an immediate immune response to the invasion of fungi. The two genes exhibited obvious upregulation on the first day of infection, which supports this theory.

We measured the expression level of the lysozyme gene *DcLys-4* (Fig 8) and found that, like *PPO*, it was significantly upregulated (*P*<0.05) by 2.81-fold on the first day of infection, 17.37-fold on the third day, and 27.00-fold on the fifth day.

Heat shock cognate 70 (*DcH70-1*, Fig 8) acts in the MAPK signaling pathway and responds to exogenous stimuli. Expression of this gene did not change significantly in the first 2 days of infection. But during the last 3 days, it was significantly upregulated (*P*<0.05) with a 7.34-fold change on the fifth day.

In the expression profile, the hypothetical protein gene *DcHP-1* also showed a large fold change of 8.13-fold on the fourth day and by 22.82-fold (Fig 8) on the fifth day. Further investigation of the importance of this gene is warranted.

## Discussion

We obtained a transcriptome database containing 84,733 unigenes of *D*. *citri* using RNA-Seq technology. This amount of data was greater than that obtained previously [26]. By searching and screening the transcriptome database, we identified 129 immunity-related unigenes. These unigenes are related to pattern recognition receptors, information transduction factors and response factors. Using the digital gene expression profile, we identified 441 differentially expressed genes in *D*. *citri* infected with *L*. *attenuatum*. Among these genes, we found the response factors such as the PPO, lysozyme, and clip-domain SP participated in cascade. However, we did not find genes related to the pattern recognition receptors, suggesting that as the final effector, response factors show fluctuating expression

The cuticle is the first barrier for insects to defend against pathogen infection, in addition to being indispensible for maintaining the shape and mobility of insects [27, 28]. The major components of the insect cuticle are chitin and cuticle protein [29]. Cuticle protein contributes much to the stress resistance, drug resistance, and immunity of insects. When an insect is suffering from adverse environment conditions, cuticle protein genes are induced to strengthen or stabilize the cuticular structure, resist the effects of adverse factors, and maintain the insect's survival [30–32]. In researching the aphid's insecticide resistance mechanism, Silva et al. found that two RR2-type cuticle protein genes are upregulated [33]. Asano et al. found that when *B*. *mori* larvae are subjected to bacterial infection, the cuticle protein gene B*mCb10* is significantly upregulated. They speculated that the gene could transmit the exogenous adverse stimulation to activate melanization [34]. He *et al*. proposed that cuticle protein may play a role in wound healing in *Anopheles gambiae* larvae and adults [35]. In this study, two cuticle protein genes showed significant upregulation upon *D*. *citri* infection by *L*. *attenuatum*. This finding provides some evidence for the role of cuticle protein in the immune defense of *D*. *citri*.

Melanization, an important aspect of the insect defense system, involves the regulation of the melanin cascade mediated by PPO [36]. Upon pathogen invasion, it activates PPO and transforms it into PO, which can transform phenolic substances into quinone intermediates, that aggregate and form melanin before enclosing, isolating, and killing pathogens. In addition, PO takes part in the processes of wound healing and skin hardening. The activation and transformation of PPO into PO is generally considered to be completed through the cascade of clip-domain SPs [37]. Gillespie *et al*. found that when *Schistocerca gregaria* was infected by *Metarhizium anisoplia*, the PPO level in the body increased while PO activity decreased, and the lysozyme level exhibited a significant decrease in comparison with the control group [11]. By knocking out the PPO gene in the mosquito *Armigeres subalbatus*, it was found that the melanization function was influenced greatly [38]. When fungal spores were injected into the insect body, the PO expression level was increased significantly [39]. We measured the expression levels of clip-domain SP and PPO in *D*. *citri* infected by *L*. *attenuatum*. On the first day of infection, both were significantly upregulated, and the fold changes in expression exceeded 35-fold by the fifth day. This indicates that these enzymes contribute much to the response against exogenous pathogenic fungi.

In the Digital Gene Expression Profiling, vitellogenin and cathepsin B with high expression levels received our attention. vitellogenin is an important source of energy for *D*. *citri*, and cathepsin B is an indispensable metabolic enzyme of lysosome. However, research on their roles in the immune response remains incomplete. Guo *et al*. showed that when *Bemisia tabaci* were fed a virus-infected plant, vitellogenin expression increased significantly [40]. Soderhall et al. demonstrated that a clotting protein belonging to the vitellogenin superfamily participates in the autologous immune defense in freshwater crayfish [41]. Raikhel et al. found that the upstream regulatory region of the vitellogenin gene of *Aedes aegypti* participates in the immune defense against pathogens [42]. Shi et al. demonstrated that vitellogenin can agglutinate erythrocytes of toad and chicken and has an inhibitory effect on various bacteria [43]. Amdma et al. showed that vitellogenin participates in the regulation of immune function and life in bees [44]. Cathepsin B enzymes can part in the processes of immune evasion [45, 46]. Futahashi et al. found upregulated expression of the cathepsin gene in many tissues of *Burkholderia*-infected *Riptortus pedestris* [47]. Zhang et al. cloned the cathepsin O gene of *B*. *mori* and detected its expression in the hemolymph of *B*. *mori* treated with *Escherichia coli*, they speculated that the enzyme participates in the immune response in the body [48]. Kocks et al. found that after immune stimulation, cysteine protease L was activated and highly expressed in lysosomes [49]. Wang et al. investigated two Cathepsin L genes of channel catfish and proved that the two genes acted in mucosal immunity [50].

In future studies, we may take advantage of the RNAi technique to verify the effect of these genes in the response of *D*. *citri* to infection of entomopathogenic fungi, and improve the fungal toxicity and field application effectiveness.

## Supporting Information

S1 Fig Total RNA extraction from *D*. *citri*. 1: Total RNA for transcriptome sequencing; 2,3: Total RNA for digital gene expression profiling.
(TIF)

S1 Table Primer pairs for real time quantitative PCR in *D*. *itri*. (DOC)
