# Supplementary figures and images for: Identification of Immunity-Related Genes in Dialeurodes citri against Entomopathogenic Fungus Lecanicillium attenuatum by RNA-Seq Analysis
Source: PLoS One. 2016 Sep 19;11(9):e0162659. doi: 10.1371/journal.pone.0162659 (PMC5028029; doi:10.1371/journal.pone.0162659)

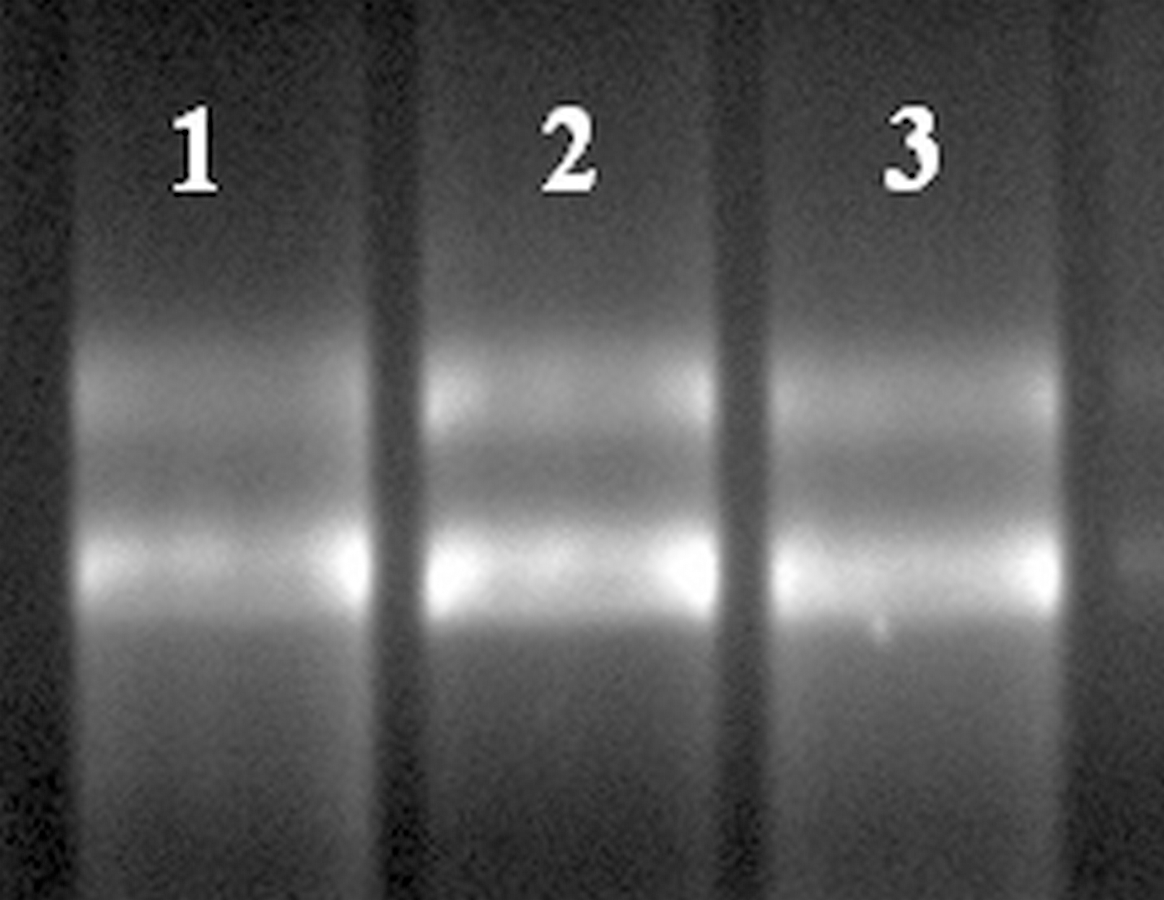

Supplement: S1 Fig — 1: Total RNA for transcriptome sequencing; 2,3: Total RNA for digital gene expression profiling. (TIF) [file pone.0162659.s001.tif]
