# Supplementary material for: Identification of Immunity-Related Genes in Dialeurodes citri against Entomopathogenic Fungus Lecanicillium attenuatum by RNA-Seq Analysis
Source: PLoS One. 2016 Sep 19;11(9):e0162659. doi: 10.1371/journal.pone.0162659 (PMC5028029; doi:10.1371/journal.pone.0162659)
Supplement: S1 Table — (DOC) [file pone.0162659.s002.doc]

***Table S1 Primer pairs for real time quantitative PCR in*** D. itri.

| **Gene name** | **Primer sequence (5′-3′)** | **Length (bp)** |
| --- | --- | --- |
| comp78104_c1 | F:GCTATCTACGCTGCCTCA | 87 |
|  | R:GCCGACTGGAACTTCACA |  |
| comp68271_c0 | F:CCTTCGGACTTCCTCTCA | 100 |
|  | R:CTCTGGTGGTGCTTGATG |  |
| comp73224_c1 | F:GCAGCGAAGAAGACTACGA | 112 |
|  | R:ACTTGCTTGGAGGATTGGAT |  |
| comp77391_c0 | F:GTAGAAGAGGAAGCGAGACT | 80 |
|  | R:GTGACAAGGAAGCCGAATAC |  |
| comp78697_c0 | F:GATTGGCTCTGTAGAGGATGA | 111 |
|  | R:CCCGCTCGTAAATGTATTTGAA |  |
| comp76462_c0 | F:CCCTACTGGCTGGCTGCTAACT | 98 |
|  | R:CGGACGGACGAGAACGGAGAAT |  |
| comp78621_c0 | F:CAATTTCAAATTCGACATCG | 101 |
|  | R:TAATCTTGTACGTGAACGGA |  |
| comp80903_c0 | F:TCCTGCCACTATGCCGCTAATCTC | 116 |
|  | R:CGTCTCCAGTTGCTCCCGAACA |  |
| comp62670_c0 | F:AATGGTTTTATAAGATAAAT | 85 |
|  | R:CACTGCCTGGACCACCTACA |  |
| comp78031_c1 | F:ACTGCCGAACCTACCTCAAGCC | 81 |
|  | R:ATCCATACTGTGGCAACCGTGA |  |
| comp79952_c0 | F:AAGCCTCGTCCTCCACTA | 76 |
|  | R:AGATTCGTCCTGAGACACCT |  |
| comp693684_c0 | F:CCGTCGTCTCCGTACTGCTTG | 90 |
|  | R:TCGAAACGAGCACGGGTAATG |  |
| comp82372_c0 | F:GGATGACGACGAAGAAGATG | 103 |
|  | R:AACCAGAGACTGAGCCTATC |  |
| α-tubulin | F:AGGATGCCGCCAATAACTACGC | 88 |
|  | R:GCCAACTTACGAATACGGTCTA |  |
| β-actin | F:GCTATGAGTTGCCTGATG | 122 |
|  | R:GTATGTGGTCTCGTGGAT |  |
